# Cohesion between pulmonary artery and bronchus after immune checkpoint inhibitor therapy in a lung cancer patient

**DOI:** 10.1111/1759-7714.13697

**Published:** 2020-10-16

**Authors:** Shinkichi Takamori, Mitsuhiro Takenoyama, Taichi Matsubara, Takatoshi Fujishita, Kensaku Ito, Masafumi Yamaguchi, Ryo Toyozawa, Takashi Seto, Tatsuro Okamoto

**Affiliations:** ^1^ Department of Thoracic Oncology National Hospital Organization Kyushu Cancer Center Fukuoka Japan; ^2^ Department of Surgery Matsuyama Red Cross Hospital, 1 Bunkyo‐cho Matsuyama Japan

**Keywords:** Non‐small cell lung cancer, programmed death‐ligand 1, surgery

## Abstract

Immunotherapy targeting programmed death‐1 or programmed death‐ligand 1 has become the standard of care for advanced non‐small cell lung cancer (NSCLC). Several recent clinical trials have investigated the efficacy of immune checkpoint inhibitors (ICIs) as neoadjuvant treatment for early NSCLC. However, the safety and feasibility of pulmonary resection after ICIs remain unclear. We herein report a patient in whom cohesion between the left main pulmonary artery and left upper bronchus was found during left upper lobectomy following neoadjuvant ICI combined with chemotherapy. After both central and peripheral sides of the left main pulmonary artery were clamped with the aim of controlling hemorrhage in case of vascular injury, the left main pulmonary artery and left upper bronchus were divided and individually cut with staplers. The thoracoscopic procedure was otherwise uneventful. The patient was discharged from our hospital with no postoperative complications. Thoracic surgeons should anticipate the possible need for management of cohesion between a pulmonary artery and bronchus in patients who have received immune checkpoint inhibitors preoperatively.

## Graphical abstract

We herein report a patient in whom cohesion between the left main pulmonary artery and left upper bronchus was found during left upper lobectomy following neoadjuvant immune checkpoint inhibitor combined with chemotherapy. The left main pulmonary artery and left upper bronchus were successfully divided and individually cut with staplers. Thoracic surgeons should anticipate the possible need for management of cohesion between a pulmonary artery and bronchus in patients who have received immune checkpoint inhibitors preoperatively.

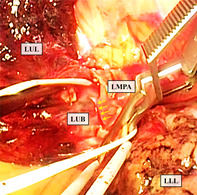

## Introduction

Immunotherapy targeting programmed death‐1 or programmed death‐ligand 1 has become the standard of care for advanced non‐small cell lung cancer.^1^, ^2^, ^3^, ^4^ A recent clinical trial reported that neoadjuvant nivolumab was associated with few adverse effects and induced major pathological responses in 45% of patients who underwent resection of early non‐small cell lung cancer.^5^ Several studies have reported finding adhesions and fibrotic changes in fissures, the chest wall, and hilum during thoracic surgery following anti‐programmed death‐1/programmed death‐ligand 1 therapy.^6^, ^7^ However, the safety and feasibility of pulmonary resection after immune checkpoint inhibitors (ICIs) remains unclear. We herein report a patient who was found to have cohesion between the left main pulmonary artery and left upper bronchus during left upper lobectomy following neoadjuvant ICI combined with chemotherapy.

## Case report

**Figure 1:**
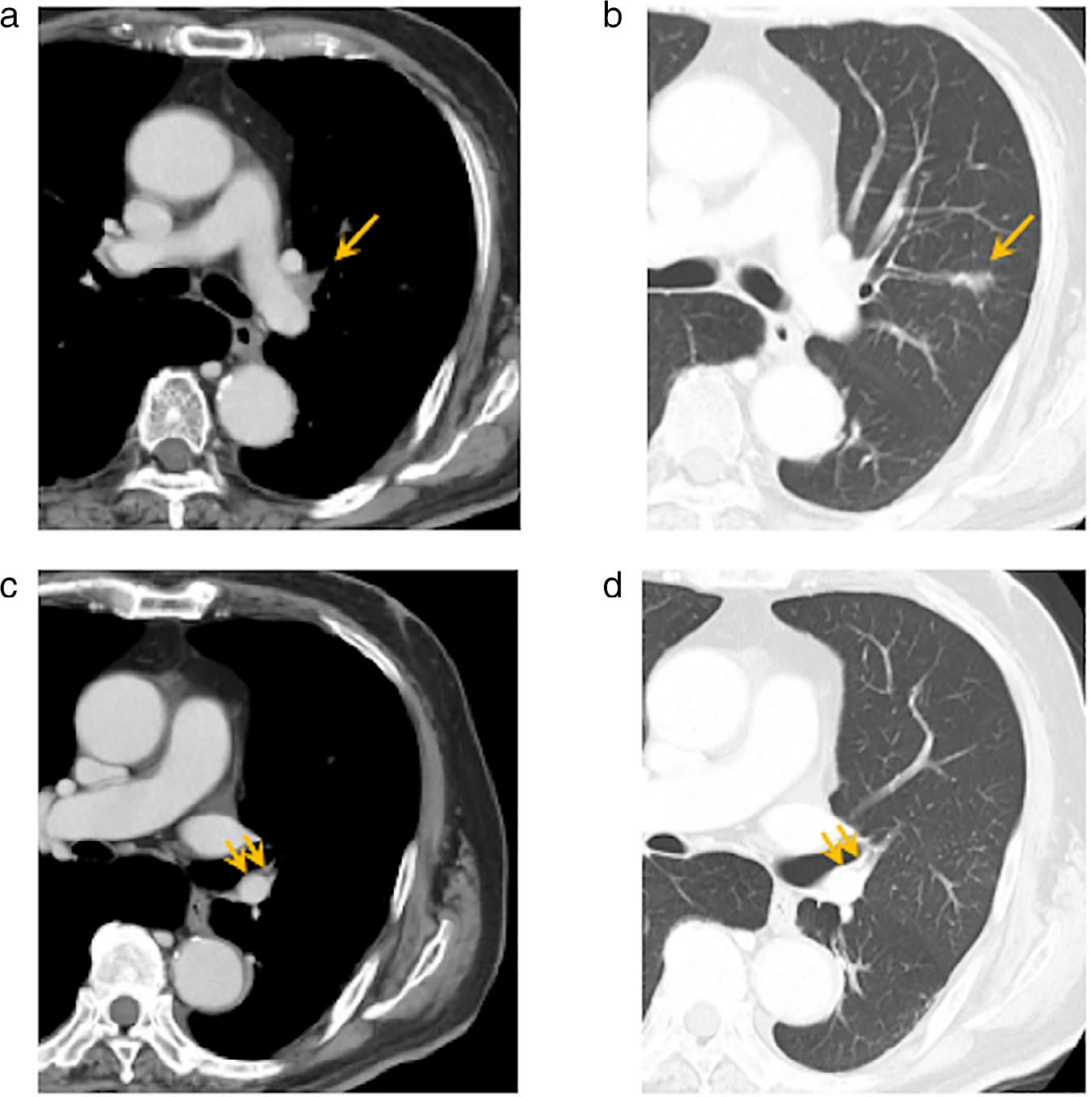
Preoperative images of computed tomography (after neoadjuvant therapy). **(a)** The single‐station N1 lymph node metastasis is shown (orange arrow). **(b)** The primary lung tumor in the left upper lobe is shown (orange arrow). **(c, d)** There were no abnormal findings between the left main pulmonary artery and the left upper bronchus (orange arrows).

**Figure 2:**
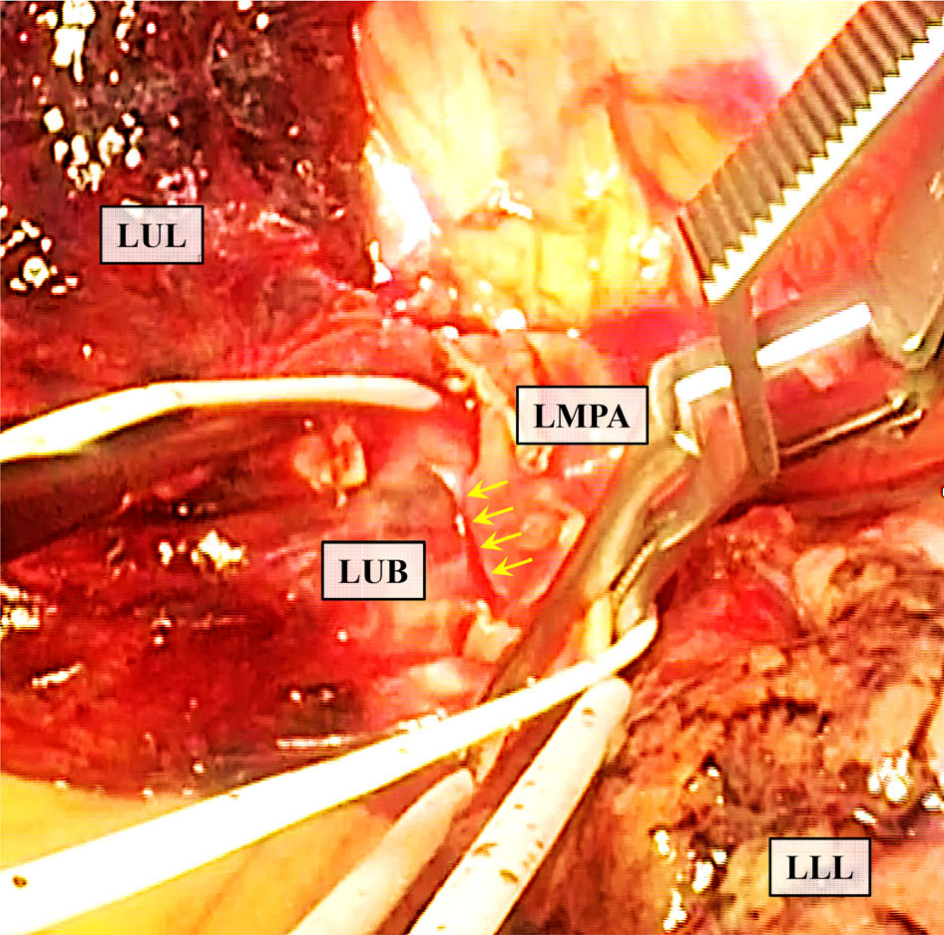
Intraoperative photograph showing cohesion between the left main pulmonary artery and the left upper lobe bronchus (yellow arrows). LLL, left lower lobe; LMPA, left main pulmonary artery; LUB, left upper bronchus; LUL, left upper lobe.

A 74‐year‐old man with a 55‐pack‐year smoking history presented to our hospital. Computed tomography (CT) revealed a mass in the left upper lobe of the lung (maximum diameter: 3.2 cm) with left hilar lymph node swelling (#10). He underwent a positron emission tomography (PET) scan and was clinically diagnosed as having left lower lobe lung cancer together with metastasis in a left hilar lymph node. Transbronchial lung biopsy resulted in a diagnosis of adenocarcinoma in the left upper lobe (cT2aN1M0, cStage IIB). He received four cycles of an ICI combined with cytotoxic chemotherapy as a participant in a clinical trial of neoadjuvant therapy (Fig 1). Four months later, he underwent left upper lobectomy and lymph node dissection (ND2a‐2). During the procedure, the left main pulmonary artery was found to be firmly fixed to the left upper lobe bronchus (Fig 2). Both central and peripheral sides of the left main pulmonary artery were clamped with the aim of controlling hemorrhage in case of vascular injury, after which the artery was separated with difficulty from the bronchus. The left main pulmonary artery and left upper bronchus were then divided and individually cut with staplers. The thoracoscopic procedure was otherwise uneventful. The patient was discharged from our hospital with no postoperative complications.

## Discussion

Here, we present a patient who following neoadjuvant ICI combined with chemotherapy, underwent left upper lobectomy. Because preoperative computed tomography (CT) examination had shown a single‐station bulky N1 lymph node metastasis (#10), we anticipated that it would be difficult to dissect the pulmonary artery from that lymph node. However, we did not expect any problem with dissection of the left main pulmonary artery because there were no adjacent lymph node metastases. In previous studies, simultaneous stapling of pulmonary vessel and bronchus have been performed because of previous surgery and/or silicotic lymphadenitis.^8^, ^9^ In the current report, there was no previous surgery or silicotic lymphadenitis. In addition, preoperative CT suggested no lymphadenopathy in the #11 area, where the cohesion was observed, and PET‐CT also showed no specific accumulation of fluorodeoxyglucose in that area. Thus, although there is limited data on similar adverse events in the prospective studies, we believe that the cohesion was not caused by lung cancer development, but by the effect of preoperative ICI. Clamping both central and peripheral sides of the left main pulmonary was necessary in case of vascular injury. We have identified only one similar previous case: Bott *et al*.^7^ reported fusion between a pulmonary artery and bronchus following administration of an ICI. Those authors reported that the truncus branch of the pulmonary artery and a bronchus were fused and that they cut both of them together with a single stapler.^7^

According to previous reports on the safety of lung resection after administration of ICIs, cohesion between bronchus and pulmonary artery was not observed in patients who received ICIs that were not in a neoadjuvant setting.^7^, ^10^ However, a recent clinical trial investigating the safety and efficacy of neoadjuvant nivolumab in NSCLC showed that 9% of patients receiving nivolumab in a neoadjuvant setting had bronchopleural fistulas.^11^ This difference may result from whether metastatic NSCLC cells from primary lesions circulate through the lymphatic capillaries around the airways, and provoke the immunological responses by ICIs. In the current case, given that the preoperative CT and PET indicated #10 lymphatic metastasis from primary adenocarcinoma, the lymphatic capillaries around #11 lymph node, where the cohesion was observed, may have had the metastatic NSCLC cells in the connective tissue sheaths around the pulmonary artery and bronchi. ICIs may provoke an antigen–antibody reaction by inhibiting the interaction of programmed death‐1 with its ligands. The resultant immune responses in the connective tissue sheaths around the pulmonary artery and bronchi may result in adhesion between them. However, this is only a hypothesis, and should be investigated in further studies. The pathological findings, especially on the surface of the left main pulmonary artery adjacent to the left upper bronchus, are crucial to discussing the pathogenesis of the cohesion between the vessel and bronchus in the current patient. However, because the patient received a neoadjuvant ICI in a clinical trial setting, we are bound by confidentiality restrictions regarding both pathological responses and the specific investigational drug administered. Nevertheless, the intraoperative findings we have reported here are extremely important for thoracic surgeons who are planning to perform lobectomy, segmentectomy, or pneumonectomy.

In conclusion, the possibility of cohesion between vessels and bronchi during thoracic surgery, as well as the attendant necessity for vascular management, should be considered in the planning of thoracic surgery in patients who have undergone immune checkpoint inhibitor and chemotherapy.

## Disclosure

None of the authors have conflicts of interest to declare in association with this study.
